# Associations of Skipping Breakfast, Lunch, and Dinner with Weight Gain and Overweight/Obesity in University Students: A Retrospective Cohort Study

**DOI:** 10.3390/nu13010271

**Published:** 2021-01-19

**Authors:** Ryohei Yamamoto, Ryohei Tomi, Maki Shinzawa, Ryuichi Yoshimura, Shingo Ozaki, Kaori Nakanishi, Seiko Ide, Izumi Nagatomo, Makoto Nishida, Keiko Yamauchi-Takihara, Takashi Kudo, Toshiki Moriyama

**Affiliations:** 1 Health and Counseling Center, Osaka University, 1-17 Machikaneyamacho, Toyonaka, Osaka 560-0043, Japan; ryoshimura@kid.med.osaka-u.ac.jp (R.Y.); k-nakanishi@wellness.hss.osaka-u.ac.jp (K.N.); ide@hacc.osaka-u.ac.jp (S.I.); iznagatomo@hacc.osaka-u.ac.jp (I.N.); mnishida@wellness.hss.osaka-u.ac.jp (M.N.); takihara@wellness.hss.osaka-u.ac.jp (K.Y.-T.); kudo@psy.med.osaka-u.ac.jp (T.K.); moriyama@wellness.hss.osaka-u.ac.jp (T.M.); 2 Department of Nephrology, Osaka University Graduate School of Medicine, 2-2-D11 Yamadaoka, Suita, Osaka 565-0871, Japan; rtomi@kid.med.osaka-u.ac.jp (R.T.); shinzawa@kid.med.osaka-u.ac.jp (M.S.); shingo.oz@kid.med.osaka-u.ac.jp (S.O.); 3 Health Promotion and Regulation, Department of Health Promotion Medicine, Osaka University Graduate School of Medicine, 1-17 Machikaneyamacho, Toyonaka, Osaka 560-0043, Japan

**Keywords:** meal frequency, breakfast skipping, lunch skipping, dinner skipping, weight gain, overweight/obesity, retrospective cohort study

## Abstract

Although multiple studies have identified skipping breakfast as a risk factor for weight gain, there is limited evidence on the clinical impact of skipping lunch and dinner on weight gain. This retrospective cohort study including 17,573 male and 8860 female university students at a national university in Japan, assessed the association of the frequency of breakfast, lunch, and dinner with the incidence of weight gain (≥10%) and overweight/obesity (body mass index ≥ 25 kg/m^2^), using annual participant health checkup data. Within the observation period of 3.0 ± 0.9 years, the incidence of ≥10% weight gain was observed in 1896 (10.8%) men and 1518 (17.1%) women, respectively. Skipping dinner was identified as a significant predictor of weight gain in multivariable-adjusted Poisson regression models for both men and women (skipping ≥ occasionally vs. eating every day, adjusted incidence rate ratios, 1.42 (95% confidence interval: 1.02–1.98) and 1.67 (1.33–2.09) in male and female students, respectively), whereas skipping breakfast and lunch were not. Similarly, skipping dinner, not breakfast or lunch, was associated with overweight/obesity (1.74 (1.07–2.84) and 1.68 (1.02–2.78) in men and women, respectively). In conclusion, skipping dinner predicted the incidence of weight gain and overweight/obesity in university students.

## 1. Introduction

Obesity, defined as a disease resulting from excess body weight, is one of the major global health burdens, along with smoking, hypertension, and diabetes [1]. Obesity is a critical lifestyle factor that increases the risk of developing cardiometabolic diseases such as hypertension [1], diabetes [2], and cardiovascular disease (CVD) [3], and increases mortality [4]. Several studies suggest that prevention of obesity in the young population is crucial. Firstly, body mass index (BMI) at a younger age predicts the occurrence of obesity in adulthood [5]; secondly, there is a stronger association between obesity and mortality at a younger age than at an older age [4,6]. One of the potential outcomes is weight gain in first-year university students, often referred to as “Freshman 15” [7]. Ethnicity, sex, dietary behavior, and physical activity were identified as major predictors of weight gain in first-year university students [8].

One of the dietary risk factors for overweight/obesity is meal frequency [9]. Multiple observational studies have reported that low eating frequency is associated with overweight/obesity [10]. Among breakfast, lunch, and dinner, the association between breakfast frequency and overweight/obesity has been the most extensively studied. The Coronary Artery Risk Development in Young Adults (CARDIA) study reported an inverse dose-dependent association between breakfast frequency (0–3, 4–6, and 7 days/week) and the incidence of obesity (BMI ≥ 30 kg/m^2^) in 3598 young adults [11]. A systematic review including 36 cross-sectional and 9 cohort studies verified that skipping breakfast increased the risk of overweight/obesity [12]. Few studies have reported the clinical impact of lunch and dinner frequency on overweight/obesity [13,14], partly because a very low percentage of adults skip dinner [15].

The aim of this retrospective cohort study was to assess the clinical impact of skipping breakfast, lunch, and dinner on weight gain and overweight/obesity in 26,433 university students within their 6-year college life.

## 2. Materials and Methods

### 2.1. Participants

**Figure 1:**
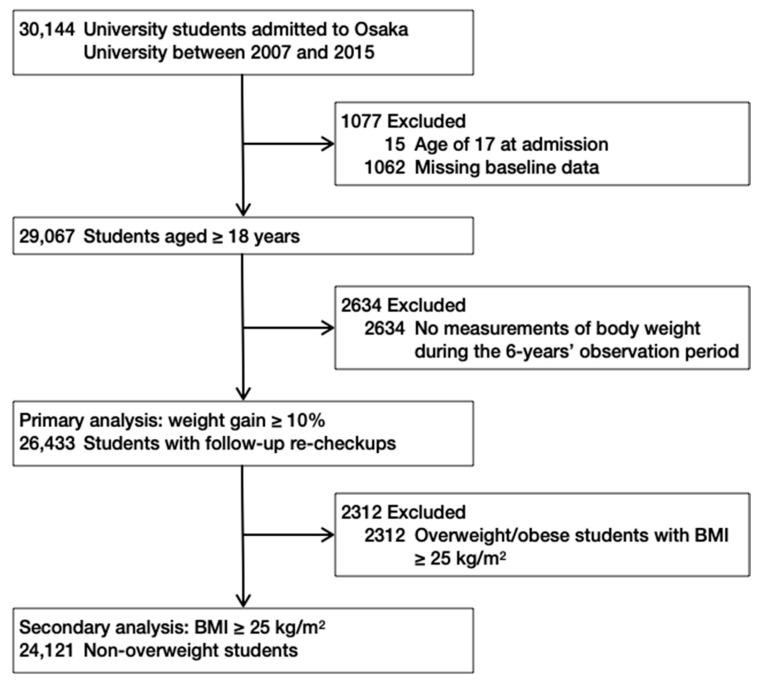
Flow diagram of inclusion and exclusion of study participants. BMI, body mass index.

Over 30,144 university students enrolled at Osaka University, one of the largest national universities in Japan, between 2007 and 2015 who underwent baseline health checkup on admission at the Osaka University Healthcare Center in April or October were eligible for inclusion in this retrospective cohort study. We excluded 15 (0.0%) students aged 17 years and 1062 students (3.5%) with missing baseline data (Figure 1). Osaka University provides annual health checkups to university students and graduate students every April or October (Figure S1). After excluding 2634 (8.7%) students who had no body weight measurements during the 6-year observation period, 26,433 (87.7%) were included in the final analysis to assess the association between the frequency of breakfast, lunch, and dinner over the year preceding admission and weight gain after admission.

The study protocol was approved by the Ethics Committee of the Health and Counseling Center at Osaka University (No. 2020-7) and the Osaka University Hospital (No. 18352). Informed consent was not obtained from all the participants according to the Japanese Ethical Guidelines for Medical and Health Research Involving Human Subjects. All the data were retrieved from the electronic database of the Health and Counseling Center, Osaka University.

### 2.2. Measurements

The baseline health checkup variables at admission included age, BMI (body weight (kg)/height^2^ (m^2^)), and questionnaires on meal frequency, dinner time, sleep duration on weekdays, smoking status, and drinking status. Questions related to these items were included in the general health questionnaires administered at the baseline health checkup. Breakfast, lunch, and dinner frequency were determined by asking the question “How often did you have breakfast/lunch/dinner over the past year?”; the possible responses were “Eating almost every day”, “Skipping occasionally”, “Skipping often”, and “Usually skipping.” Dinner time was determined by asking the question “What time did you have dinner?”; the four possible answers were as follows: “Before 7 PM”, “7–9 PM”, “9–11 PM”, and “After 11 PM.” Sleep duration on weekdays was determined by asking the question “How long do you sleep on weekdays?”; the possible answers were “< 5 h”, “5–6 h”, “6–7 h”, “7–8 h”, and “≥8 h.” Smoking status was determined by asking the question “Do you smoke?”; the possible answers were “I do not smoke”, “I quitted smoking”, “I would like to quit smoking”, and “I smoke.” Drinking status was determined by asking the question “Do you drink alcohol?”; the possible answers were “I do not drink”, “I drink occasionally”, “I drink 1 day/week”, “I drink 2–3 days/week”, and “I drink ≥4 days/week.”

The outcome measures of the present study were weight gain ≥10% [16] in all students and overweight/obesity (BMI ≥ 25 kg/m^2^) [17] in students with a BMI of <25 kg/m^2^. Observational time was defined as the period from the health checkup at admission to (i) the incidence of each outcome or (ii) the last body weight measurement at the annual Osaka University health checkups within 6 years (=2192 days) of the baseline checkup before April 2019, whichever came first.

To assess the degree to which the baseline dinner frequencies reflected the dinner frequency during the observation period, the answers to “How often did you have dinner during the past year?” at the annual health checkups for university students 1 year (365 days) and 3 years (1095 days) after the baseline checkup, with a window period of ±182 days, were retrieved.

### 2.3. Statistical Analysis

Because of the small number of students skipping dinner often (men, n = 20 (0.1%); women, n = 32 (0.4%)) and usually (men, n = 4 (0.0%); women, n = 11 (0.1%)), dinner frequency was divided into two groups: eating every day and skipping dinner ≥ occasionally. Similarly, students skipping lunch often (men, n = 76 (0.4%); women, n = 20 (0.2%)), and usually (men, n = 21 (0.1%); women, n = 7 (0.1%)) were categorized as skipping lunch ≥ occasionally. Breakfast frequency was classified as eating every day, skipping occasionally, and skipping ≥ often, including skipping often (men, n = 663 (3.8%); women, n = 209 (2.4%)) and usually (men, n = 402 (2.3%); women, n = 76 (0.9%)). Because 17,476 (99.4%) men and 8847 (99.9%) women answered “I do not smoke”, subjects were categorized into non-smokers with “I do not smoke” and smokers with other answers. Drinking status was classified into two categories: non-drinkers who answered “I do not drink” (men, n = 15,915 (90.6%); women, n = 8442 (95.3%)) and drinkers who answered “I drink occasionally” (men, n = 1377 (7.8%); women, n = 378 (4.3%)), “I drink 1 day/week” (men, n = 128 (0.7%); women, n = 21 (0.2%)), “I drink 2–3 day/week” (men, n = 127 (0.7%); women, n = 16 (0.2%)), and “I drink ≥4 days/week” (men, n = 26 (0.1%); women, n = 3 (0.0%)). The clinical characteristics of the different meal frequency categories were compared using the Student’s t-test, analysis of variance, and the chi-squared test, as appropriate.

The cumulative probability of the incidence of weight gain ≥10% and BMI ≥ 25 kg/m^2^ was calculated using the Kaplan–Meier method and compared using the log-rank test among the categories of meal frequency. To assess the association between the baseline meal frequency and the incidence of each outcome, unadjusted and multivariable-adjusted incidence rate ratios (IRR) with the corresponding 95% confidence interval (CI) of each meal frequency category were calculated using Poisson regression models. Model 1 was an unadjusted model including frequency of each meal as an independent variable. In model 2, the association between frequency of each meal and the outcomes were assessed after adjusting for admission year (2007, 2008, 2009, 2010, 2011, 2012, 2013, 2014, and 2015), age (18, 19, 20, and ≥21 years), BMI (kg/m^2^), smoking status (non-smokers and smokers), drinking status (non-drinkers and drinkers), sleep duration [18] on weekdays (<5, 5–6, 6–7, 7–8, and ≥8 h), and dinner time [19] (before 7 PM, 7–9 PM, 9–11 PM, and after 11 PM). Model 3 assessed the independent associations between breakfast, lunch, and dinner frequency with the outcomes after adjusting for each other. Robust (Huber–White) sandwich-based standard errors were used to validate inferences for estimates in Poisson regression models. Appropriateness of Poisson regression models was tested with a goodness-of-fit test using the deviance statistic.

To evaluate an association between the baseline dinner frequency and the dinner frequency during the observation period, reproducibility of the baseline dinner frequency with the dinner frequency 1 and 3 years after admission was assessed using the Gwet’s AC1 coefficient, which was calculated using Stata’s kappaetc command [20]. Reproducibility with Gwet’s AC1 coefficients of <0.00, 0.00–0.20, 0.21–0.40, 0.41–0.60, 0.61–0.80, or 0.81–1.00 was regarded as poor, slight, fair, moderate, substantial, or almost perfect, respectively [21].

To identify the characteristics of students who kept skipping dinner ≥ occasionally, the baseline characteristics were compared between students who kept skipping dinner ≥ occasionally at admission and 1 year after admission and those who skipped dinner ≥ occasionally at admission but ate dinner every day 1 year after admission using the Student’s t-test and the chi-squared test.

Continuous variables were expressed as the mean ± standard deviation, while categorical variables were expressed as numbers and proportions. A *p* value <0.05 was considered significant. All statistical analyses were performed using Stata version 16.1 (Stata Corp, College Station, TX, USA).

## 3. Results

Baseline characteristics of 17,573 male students are listed in Table 1. Compared with the 17,307 (98.5%) male students who ate dinner every day, those who skipped dinner ≥ occasionally (n = 266 (1.5%)) were likely to be older and more overweight, have shorter sleep duration and higher prevalence of smokers and drinkers, skip other meals more frequently, and eat dinner later (*p* < 0.05). With regard to the frequency of breakfast and lunch frequency, similar trends were observed in male students (*p* < 0.05), except for BMI (Tables S1 and S2). Of the 8860 female students, those who skipped dinner ≥ occasionally (n = 358 94.0%)), were likely to be older and more overweight, have shorter sleep duration and higher prevalence of smokers and drinkers, and skip other meals more frequently than the 8502 (96.0%) female students who ate dinner every day (*p* < 0.05) (Table 2). Female students who skipped breakfast and lunch were likely to be older, have longer sleep duration and higher prevalence of drinkers, skip other meals, and eat dinner later than those who ate breakfast and lunch every day (*p* < 0.05) (Tables S3 and S4).

**Figure 2:**
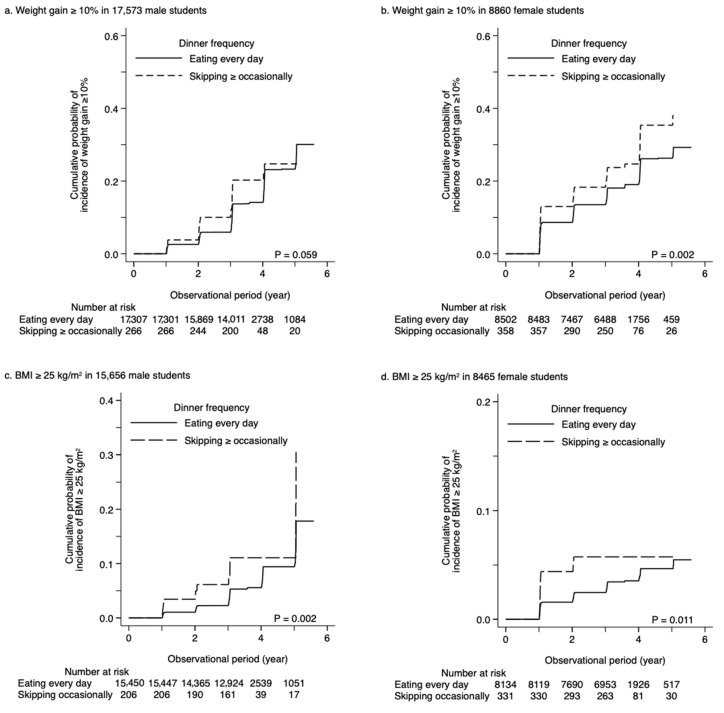
Dinner frequency and the cumulative probabilities of the incidence of ≥10% weight gain (**a**,**b**) and BMI ≥ 25 kg/m^2^ (**c**,**d**) in male (**a**,**c**) and female (**b**,**d**) students. BMI, body mass index.

**Figure 3:**
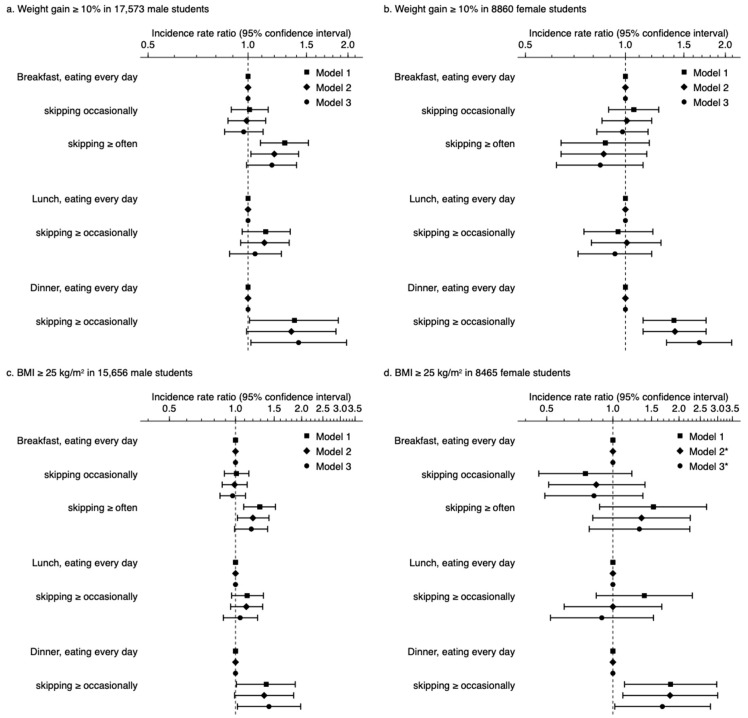
Meal frequency and incidence of ≥10%weight gain (**a**,**b**) and body mass index (BMI) ≥ 25 kg/m^2^ (**c**,**d**). BMI, body mass index. Model 1, unadjusted. Model 2, adjusted for admission year (2007, 2008, 2009, 2010, 2011, 2012, 2013, 2014, and 2015), age (18, 19, 20, and ≥21 years), BMI (kg/m^2^), smoking status (non-smokers and smokers), drinking status (non-drinkers and drinkers), dinner time (before 7 PM, 7–9 PM, 9–11 PM, and after 11 PM), and sleep duration on weekdays (<5, 5–6, 6–7, 7–8, and ≥8 h). Model 3, adjusted for covariates in model 2 and frequencies of breakfast, lunch, and dinner (e.g., breakfast frequency was adjusted for lunch and dinner frequencies). * Not adjusted for smokers because of no incidence of the outcome in smokers (n = 13).

During the observation period of 3.0 ± 0.9 years, the incidence of ≥10% weight gain was observed in 1857 (10.7%) and 39 (14.7%) male students who ate dinner every day and skipped dinner ≥ occasionally, respectively (*p* = 0.040) (Table 1). Male students skipping dinner ≥ occasionally were likely to have a higher cumulative probability of weight gain than those eating dinner every day, although the difference between these groups was at a marginally significant level (*p* = 0.059) (Figure 2a). Considering breakfast frequency, male students skipping breakfast ≥ often had a higher risk of ≥10% weight gain than those eating breakfast every day and skipping breakfast occasionally (*p* = 0.029) (Figure S2a). By contrast, no significant difference was observed between male students eating lunch every day and those skipping lunch ≥ occasionally (*p* = 0.222) (Figure S3a). Unadjusted Poisson regression models showed that dinner frequency (IRR (95% CI) of skipping ≥ occasionally vs. eating every day: 1.38 (1.01–1.87)) and breakfast frequency (IRR (95% CI) of eating every day, skipping occasionally, and skipping ≥ often: 1.00 (reference), 1.02 (0.89–1.16), and 1.29 (1.10–1.52), respectively) were significantly associated with ≥10% weight gain, while there was no association between lunch frequency and ≥10% weight gain (IRR (95% CI) of skipping ≥ occasionally vs. eating every day: 1.14 (0.97, 1.35)) (Figure 3a, Model 1). Even after multivariable adjustment for clinically relevant factors, male students skipping dinner ≥ occasionally (1.42 (1.02–1.98)) were at a higher risk of ≥ 10% weight gain than those eating dinner, whereas those skipping breakfast were not (1.00 (reference), 0.97 (0.85–1.11), and 1.18 (0.99–1.40), respectively) and breakfast every day, respectively (Figure 3a, Model 3).

Of the 8502 female students eating dinner every day and the 358 female students skipping dinner ≥ occasionally, ≥10% weight gain was observed in 1436 (16.9%) and 82 (22.9%) students, respectively (Table 2). The cumulative probability of the incidence of ≥10% weight gain was significantly higher in the female students skipping dinner ≥ occasionally than in those eating every day (*p* = 0.002) (Figure 2b); by contrast, no significant difference was observed in breakfast (*p* = 0.464) (Figure S2b) and lunch frequencies (*p* = 0.677) (Figure S3b). In unadjusted Poisson regression models, dinner frequency was significantly associated with ≥10% weight gain (1.40 (1.13–1.75)), whereas breakfast frequency (1.00 (reference), 1.06 (0.89–1.26), and 0.87 (0.64–1.18)) or lunch frequency (0.95 (0.75–1.21)) were not associated with ≥10% weight gain (Figure 3b, Model 1). Multivariable-adjusted models showed a significant association between dinner frequency and ≥10% weight gain (1.67 (1.33–2.09)); however, breakfast frequency (1.00 (reference), 0.98 (0.82, 1.17), and 0.84 (0.62, 1.13)) and lunch frequency (0.93 (0.72–1.20)) were not associated with ≥10% weight gain (Figure 3b, Model 3).

After excluding 1917 (10.9%) male and 395 (4.5%) female students with BMI ≥ 25 kg/m^2^, we assessed the association between meal frequency and the incidence of overweight/obesity (BMI ≥ 25 kg/m^2^) in students with a BMI of <25 kg/m^2^. The cumulative probability of male and female students skipping dinner ≥ occasionally was significantly higher than of those eating dinner every day (*p* = 0.002 and 0.011 in male and female students, respectively) (Figure 2c,d). No significant association was observed between breakfast and lunch frequency in male students (*p* = 0.569 and 0.187 for breakfast and lunch frequency, respectively) (Figures S2c and S3c) and female students (*p* = 0.154 and 0.201, respectively) (Figures S2d and S3d). In multivariate-adjusted Poisson regression models, dinner frequency was identified as a significant predictor of BMI ≥ 25 kg/m^2^ (male students, 1.74 (1.07–2.84); female students, 1.68 (1.02–2.78)), while breakfast and lunch frequencies were not (Figure 3c,d, Model 3).

**Figure 4:**
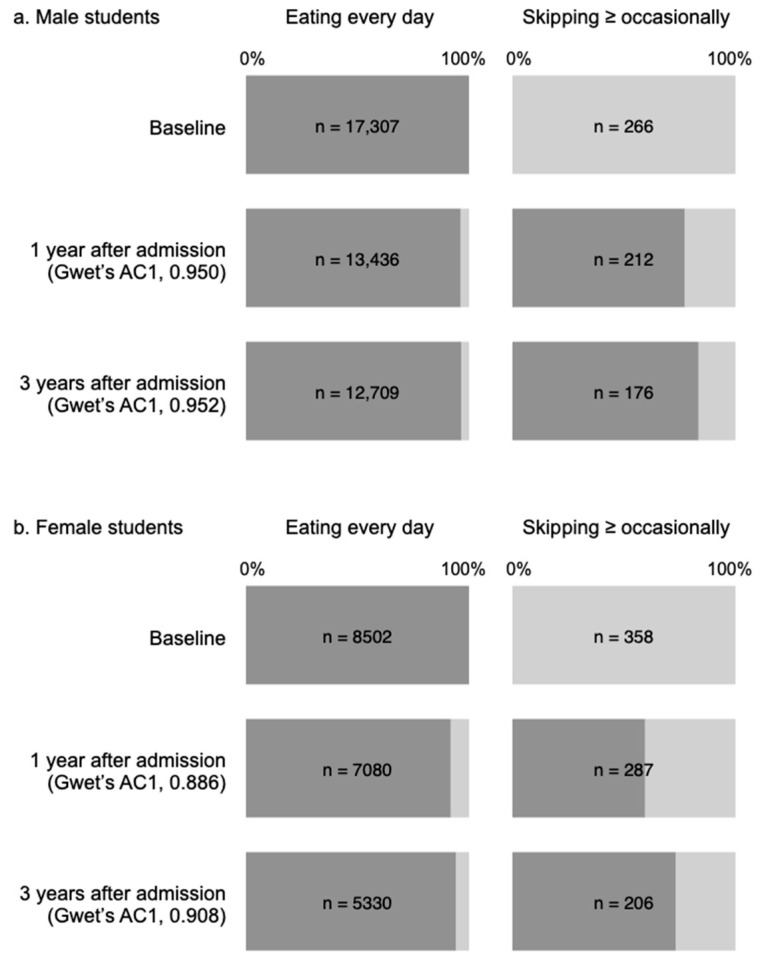
Baseline dinner frequency and dinner frequency 1 and 3 years after admission in male (**a**) and (**b**) female students. Dark and pale gray bars indicate the proportion of eating dinner every day and skipping dinner ≥ occasionally, respectively. In both male and female students, baseline dinner frequency reflected dinner frequency 1 and 3 years after admission almost perfectly (Gwet’s AC1 coefficient > 0.8).

To assess how strongly baseline dinner frequency reflected dinner frequency during the observation period, dinner frequency 1 and 3 years after admission was assessed (Figure 4). Of the 17,307 male students with baseline dinner frequency of eating every day, 13,436 (77.6%) and 12,709 (73.4%) students answered to the question of dinner frequency at their annual health checkups 1 and 3 years after admission, respectively. The vast majority of them kept eating dinner every day 1 and 3 years after admission (96.3% and 96.5%, respectively). Of the 266 male students with baseline dinner frequency of skipping ≥ occasionally, 212 (79.7%) and 176 (66.2%) gave answers at their annual health checkups 1 and 3 years after admission. Their proportion of skipping dinner ≥ occasionally 1 and 3 years after admission was 22.6% and 16.5%, which was substantially higher than that of the male students with baseline dinner frequency of eating every day (3.7% and 3.5%). The Gwet’s AC1 coefficient suggested almost perfect reproducibility of dinner frequency 1 and 3 years after submission in male students (Gwet’s AC1 coefficient 1 and 3 years after admission = 0.950 and 0.952, respectively). Similarly, almost perfect reproducibility was observed in female students (Gwet’s AC1 coefficient 1 and 3 years after admission = 0.886 and 0.908). In the female students with baseline dinner frequency of eating every day, the proportion of skipping dinner ≥ occasionally 1 and 3 years after admission was 8.1% and 5.8%, whereas that was 40.4% and 26.7% in those with the baseline dinner frequency of skipping ≥ occasionally.

To characterize the students who keep dinner frequency of skipping ≥ occasionally within 1 year of the observation period, baseline characteristics were compared between the 48 male students who kept skipping dinner ≥ occasionally at admission and 1 year after admission and the 164 male students who skipped dinner ≥ occasionally at admission but ate dinner every day 1 year after admission (Table S5). The male students who kept skipping dinner ≥ occasionally had a higher level of body weight and BMI and higher prevalence of smokers (*p* < 0.05). In contrast, no significant difference was observed between the 116 female students who kept skipping dinner ≥ occasionally and the 171 female students who skipped dinner ≥ occasionally at admission but ate dinner every day 1 year after admission (Table S6).

## 4. Discussion

This retrospective large cohort study revealed that skipping dinner was significantly associated with ≥10% weight gain and overweight/obesity (BMI ≥ 25 kg/m^2^) in both male and female students. These results suggest that skipping dinner, which was much less prevalent than skipping breakfast, has a stronger association with weight gain and overweight/obesity than skipping breakfast. The strengths of the present study are as follows: cohort study design with the mean observation period of 3 years; large sample size, which allowed the assessment of the clinical impact of low prevalence of skipping dinner; and two outcomes (≥10% weight gain and BMI ≥ 25 kg/m^2^).

Few studies have previously reported the association between skipping dinner and overweight/obesity. An Iranian cross-sectional study, the Childhood and Adolescence Surveillance and Prevention of Adult Non-communicable Disease (CASPIAN-III) study, including 5642 school students aged 10–18 years reported that students who skipped dinner had a significantly high prevalence of overweight/obesity [14]. A Spanish cross-sectional study including 16,929 men and 18,045 women aged 25–64 years showed that women who skipped dinner had a significantly higher prevalence of obesity (BMI ≥ 30 kg/m^2^), while there was no increased prevalence of obesity among the men who skipped dinner [13]. This retrospective cohort study demonstrated that skipping dinner predicted the incidence of weight gain and overweight/obesity in both the 17,573 male and the 8860 female university students. This is consistent with a 1946 British birth cohort including 1416 young adults aged 36 years reporting a dose-dependent association between the irregularity of energy intake at dinner at the age of 36 years and overweight/obesity at the age of 53 years [22]. Compared to the students who ate dinner every day, those with lower frequency of dinner were considered to have higher irregularity of energy intake at dinner. Therefore, this dose-dependent association strongly suggested that skipping dinner predicted the incidence of overweight/obesity. Although the 1946 British birth cohort also showed a significant dose-dependent association between the irregularity of energy intake at breakfast at the age of 36 years and overweight/obesity at the age of 53 years, irregularities of energy intake at breakfast and dinner were not included in the single multivariable logistic regression model; therefore, whether breakfast and dinner were independently associated with overweight/obesity remained unknown. The present study clarified that skipping dinner was independently associated with ≥ 10% weight gain and overweight/obesity in both male and female students, while skipping breakfast was not, suggesting a greater clinical impact of dinner on weight gain compared with breakfast.

One of the plausible mechanisms for the association between skipping dinner and weight gain may be an excess of energy intake due to upregulation of appetite after skipping dinner, leading to high total energy intake. Regarding breakfast, a cross-sectional study using 24-h dietary recall for two days reported that Brazilian adults who skipped breakfast at least 5 days/week had significantly higher total energy intake than those who did not [23]. Only a few cross-sectional studies reported an association between skipping dinner and total calorie intake. The National Health and Nutrition Examination Survey (NHANES) including 23,488 adults aged ≥18 years in the US [24] and a cross-sectional study including 275 nursing college students in Japan [25] showed an association between skipping dinner and low total energy intake. Differences in definitions of skipping breakfast/dinner, measurements of total calorie intake, and participants’ characteristics might have contributed to the different associations between skipping breakfast/dinner and total energy intake in these previous studies. The effect of skipping dinner on total energy should be examined in more detail.

Another potential candidate for a link between skipping dinner and weight gain may be low diet quality. The NHANES showed that skipping dinner led to a deterioration in the healthy eating index (HEI) [24], the index used to measure diet quality that predicts CVD, cancer, and all-cause mortality [26]. The Multi-ethnic Study of Atherosclerosis (MESA) reported that the baseline HEI level was inversely associated with BMI 18 months after the baseline visit, suggesting that low diet quality was a risk factor for weight gain [27]. Among the components of HEI, skipping dinner significantly reduced intake of vegetables and seafood/plant proteins compared with skipping breakfast [24]. Because a recent systematic review clarified that low vegetable intake and fish intake were associated with weight gain [28], the greater impact of skipping dinner on weight gain may be due to the larger decrease in vegetable and seafood/plant protein intake compared with skipping breakfast. Chronotype may be another plausible factor contributing to the association between skipping dinner and weight gain. A Japanese cross-sectional study including 3304 mainly female university students showed that skipping dinner was more prevalent in female students who had a later sleep midpoint [29]. Because women with an evening chronotype are prone to weight gain [30], weight gain among university students who skipped dinner might be confounded by evening chronotype. Further studies are required to assess the mechanism underlying the association between skipping dinner and weight gain.

The present study has several limitations. Firstly, it included university students aged 18–19 years mainly at a single national university in Japan. The generalizability of our results should be assessed in different cohorts. Secondly, a dose-dependent association between dinner frequency and weight gain was not assessed, because dinner frequency was determined based on a simple question with only four possible answers: “Eating almost every day”, “Skipping occasionally”, “Skipping often”, and “Usually skipping.” After asking how many times students eat dinner per week strictly, the dose-dependent association between the frequency of dinner and weight gain should be assessed in more detail. Thirdly, the association between skipping dinner and weight gain may be confounded by unmeasured factors. For example, a previous cohort study suggested that depression modified the association between evening chronotype and weight gain [23]. Because individuals with an evening chronotype were prone to skipping dinner [23], depression may have also affected the association between skipping dinner and weight gain in this study. Perceived stress might be another potential confounder. A previous cross-sectional study reported that college students who skipped dinner had a higher level of perceived stress [31]. Because some longitudinal studies identified the perceived stress as a predictor of weight gain and obesity [32,33], the association of skipping dinner with weight gain and overweight/obesity was confounded by the perceived stress. Additionally, low physical activity might affect the association of skipping dinner with weight gain and overweight/obesity. Several cross-sectional studies reported that skipping dinner was associated with low physical activity [14,34], which predicted weight gain and overweight/obesity [35,36]. The clinical impact of skipping dinner on weight gain and overweight/obesity should be examined in more well-designed studies. Fourthly, breakfast frequency was not associated with weight gain and overweight/obesity in the present study, contrary to the previous studies. One of the potential reasons for these conflicting results might be the shorter observation period (3.0 ± 0.9 years) of the present study than of the previous studies, including the Childhood Determinants of Adult Health (CDAH) study (5.0 ± 0.3 years) [37], the Adventist Health Study 2 (7.4 ± 1.2 years) [38], the Health Professionals Follow-up study (10 years) [39], and the CARDIA study (18 years) [11]. The long-term impact of skipping dinner on weight gain and overweight/obesity should be verified. Short sleep duration might be another possible reason for no significant impact of skipping breakfast on weight gain and overweight/obesity. European and Brazilian cross-sectional studies suggested that skipping breakfast was associated with BMI in adolescents with sleep duration ≥8 h/day, whereas their association was blunted in those with sleep duration <8 h [40]. Because the present study included 2.4% male and 1.3% female students who had sleep duration ≥ 8 h, skipping breakfast might not be associated with weight gain and overweight/obesity. Fifthly, a large number of students who skipped dinner ≥ occasionally over the past year of admission ate dinner almost every day during the observation period (Figure 4). Given that skipping dinner was associated with weight gain and overweight/obesity, this switching from skipping dinner ≥ occasionally to eating every day blunted the impact of skipping dinner. The true association of skipping dinner with weight gain and overweight/obesity might be stronger than that observed in the present study.

## 5. Conclusions

The present retrospective cohort study identified skipping dinner as a significant predictor of weight gain and overweight/obesity. These results suggest that dinner frequency may be a critical lifestyle factor for the prevention of obesity in addition to breakfast frequency. However, the clinical impact of dinner frequency should be clarified in further studies.

## Figures and Tables

**Table 1:** Baseline characteristics, observation period, and outcome incidences of 17,573 male students stratified by dinner frequency.

Dinner frequency	All	Eating Every Day	Skipping ≥Occasionally	*p*
Number	17,573	17,307	266	
Baseline characteristics at admission				
Age, 18 years, n (%)	11,342 (64.5)	11,228 (64.9)	114 (42.9)	<0.001
19	5625 (32.0)	5508 (31.8)	117 (44.0)	
20	387 (2.2)	367 (2.1)	20 (7.5)	
21–60	219 (1.2)	204 (1.2)	15 (5.6)	
Height (cm)	171.3 ± 5.7	171.3 ± 5.7	171.2 ± 5.6	0.763
Body weight, kg	63.4 ± 9.4	63.3 ± 9.3	67.1 ± 12.8	<0.001
BMI, kg/m^2^	21.6 ± 2.9	21.6 ± 2.9	22.9 ± 4.1	<0.001
<25.0 kg/m^2^, n (%)	15,656 (89.1)	15,450 (89.3)	206 (77.4)	<0.001
≥25.0	1917 (10.9)	1857 (10.7)	60 (22.6)	
Smokers, n (%)	97 (0.6)	84 (0.5)	13 (4.9)	<0.001
Drinkers, n (%)	1658 (9.4)	1596 (9.2)	62 (23.3)	<0.001
Sleep duration, <5 h, n (%)	547 (3.1)	532 (3.1)	15 (5.6)	<0.001
5–6	5604 (31.9)	5494 (31.7)	110 (41.4)	
6–7	8473 (48.2)	8375 (48.4)	98 (36.8)	
7–8	2521 (14.3)	2485 (14.4)	36 (13.5)	
≥8	428 (2.4)	421 (2.4)	7 (2.6)	
Breakfast, eating every day, n (%)	14,233 (81.0)	14,115 (81.6)	118 (44.4)	<0.001
Skipping occasionally	2275 (12.9)	2170 (12.5)	105 (39.5)	
Skipping ≥ often	1065 (6.1)	1022 (5.9)	43 (16.2)	<0.001
Lunch, eating every day, n (%)	16,448 (93.6)	16,327 (94.3)	121 (45.5)	<0.001
Skipping ≥ occasionally	1125 (6.4)	980 (5.7)	145 (54.5)	
Dinner time, before 7 PM, n (%)	3255 (18.5)	3201 (18.5)	54 (20.3)	<0.001
7–9 PM	11,453 (65.2)	11,310 (65.3)	143 (53.8)	
9–11 PM	2681 (15.3)	2624 (15.2)	57 (21.4)	
After 11 PM	184 (1.0)	172 (1.0)	12 (4.5)	
Observation period and outcome incidences				
Observation period (years)	3.0 ± 0.9	3.0 ± 0.9	3.0 ± 1.0	0.704
Weight gain ≥10%, n (%)	1896 (10.8)	1857 (10.7)	39 (14.7)	0.040
IR per 1000 PY (95% CI)	36.1 (34.5, 37.8)	35.9 (34.3, 37.6)	49.4 (36.1, 67.6)	
BMI ≥ 25 kg/m^2^, n (%)	671 (4.3)	653 (4.2)	18 (8.7)	0.001
IR per 1000 PY (95% CI)	14.1 (13.1, 15.2)	13.9 (12.9, 15.0)	29.0 (18.3, 46.0)	

Mean ± standard deviation; BMI, body mass index; CI, confidence interval, IR, incidence rate; PY, person-year.

**Table 2:** Baseline characteristics, observation period, and outcome incidences of 8860 female students stratified by dinner frequency.

Dinner frequency	All	Eating Every Day	Skipping ≥Occasionally	*p*
Number	8860	8502	358	
Baseline characteristics at admission				
Age, 18 years, n (%)	6537 (73.8)	6336 (74.5)	201 (56.1)	<0.001
19	2052 (23.2)	1926 (22.7)	126 (35.2)	
20	160 (1.8)	145 (1.7)	15 (4.2)	
21–60	111 (1.3)	95 (1.1)	16 (4.5)	
Height, cm	158.4 ± 5.2	158.4 ± 5.2	158.7 ± 5.1	0.250
Body weight, kg	51.5 ± 6.8	51.4 ± 6.8	53.1 ± 7.0	<0.001
BMI, kg/m^2^	20.5 ± 2.4	20.5 ± 2.4	21.1 ± 2.4	<0.001
<25.0 kg/m^2^, n (%)	8465 (95.5)	8134 (95.7)	331 (92.5)	0.004
≥25.0	395 (4.5)	368 (4.3)	27 (7.5)	
Smokers, n (%)	13 (0.1)	11 (0.1)	2 (0.6)	0.038
Drinkers, n (%)	418 (4.7)	367 (4.3)	51 (14.2)	<0.001
Sleep duration, <5 h, n (%)	264 (3.0)	246 (2.9)	18 (5.0)	0.003
5–6	3159 (35.7)	3018 (35.5)	141 (39.4)	
6–7	4209 (47.5)	4070 (47.9)	139 (38.8)	
7–8	1109 (12.5)	1057 (12.4)	52 (14.5)	
≥8	119 (1.3)	111 (1.3)	8 (2.2)	
Breakfast, eating every day, n (%)	7793 (88.0)	7538 (88.7)	255 (71.2)	<0.001
Skipping occasionally	782 (8.8)	707 (8.3)	75 (20.9)	
Skipping ≥ often	285 (3.2)	257 (3.0)	28 (7.8)	
Lunch, eating every day, n (%)	8451 (95.4)	8178 (96.2)	273 (76.3)	<0.001
Skipping ≥ occasionally	409 (4.6)	324 (3.8)	85 (23.7)	
Dinner time, before 7 PM, n (%)	2288 (25.8)	2185 (25.7)	103 (28.8)	0.111
7–9 PM	5611 (63.3)	5405 (63.6)	206 (57.5)	
9–11 PM	920 (10.4)	873 (10.3)	47 (13.1)	
After 11 PM	41 (0.5)	39 (0.5)	2 (0.6)	
Observation period and incidence of outcomes				
Observation period (years)	2.9 ± 1.0	2.9 ± 1.0	2.8 ± 1.1	0.708
Weight gain ≥10%, n (%)	1518 (17.1)	1436 (16.9)	82 (22.9)	0.041
IR per 1000 PY (95% CI)	58.1 (55.3, 61.1)	57.2 (54.3, 60.2)	80.3 (64.7, 99.7)	
BMI ≥ 25 kg/m^2^, n (%)	266 (3.1)	248 (3.0)	18 (5.4)	0.015
IR per 1000 PY (95% CI)	10.0 (8.9, 11.3)	9.7 (8.6, 11.0)	17.8 (11.2, 28.2)	

Mean ± standard deviation; BMI, body mass index; CI, confidence interval, IR, incidence rate; PY, person-year.

## Data Availability

The data presented in this study are available on request from the corresponding author. The data are not publicly available because they were not collected originally for researches.

## Supplementary Materials

The following are available online at https://www.mdpi.com/2072-6643/13/1/271/s1, Table S1: Baseline characteristics, observation period, and outcome incidences of 17,573 male students stratified by breakfast frequency; Table S2: Baseline characteristics, observation period, and incidence of outcomes of 17,573 male students stratified by lunch frequency; Table S3: Baseline characteristics, observation period, and outcome incidences of 8860 female students stratified by breakfast frequency; Table S4: Baseline characteristics, observation period, and outcome incidences of 8860 female students stratified by lunch frequency; Table S5: Baseline characteristics of 212 male students with baseline dinner frequency of skipping ≥ occasionally who underwent annual health checkup 1 year after admission; Table S6: Baseline characteristics of 288 female students with baseline dinner frequency of skipping ≥ occasionally who underwent annual health checkup 1 year after admission; Figure S1: Annual health checkups during the observation period in the students admitted in April (n = 29,985) and October (n = 159).; Figure S2: Breakfast frequency and the cumulative probabilities of the incidence of weight gain ≥ 10% (a,b) and BMI ≥ 25 kg/m^2^ (c,d) in male (a,c) and female (b,d) students; Figure S3: Lunch frequency and the cumulative probabilities of the incidence of weight gain ≥ 10% (a,b) and BMI ≥ 25 kg/m^2^ (c,d) in male (a,c) and female (b,d) students.
