# Supplementary material for: Associations of Skipping Breakfast, Lunch, and Dinner with Weight Gain and Overweight/Obesity in University Students: A Retrospective Cohort Study
Source: Nutrients. 2021 Jan 19;13(1):271. doi: 10.3390/nu13010271 (PMC7832851; doi:10.3390/nu13010271)
Supplement: Supplementary file 1 [file nutrients-13-00271-s001.pdf]

**Supplementary materials**

**Table S1.** Baseline characteristics, observational period, and outcome incidences of 17,573 male students stratified by breakfast frequency.
**Table S2.** Baseline characteristics, observational period, and incidence of outcomes of 17,573 male students stratified by lunch frequency.
**Table S3.** Baseline characteristics, observational period, and outcome incidences of 8860 female students stratified by breakfast frequency.
**Table S4.** Baseline characteristics, observational period, and outcome incidences of 8860 female students stratified by lunch frequency.
**Table S5.** Baseline characteristics of 212 male students with baseline dinner frequency of skipping ≥ occasionally who underwent annual health checkup 1 year after admission.
**Table S6.** Baseline characteristics of 288 female students with baseline dinner frequency of skipping ≥ occasionally who underwent annual health checkup 1 year after admission.
**Figure S1**. Annual health checkups during the observational period in students admitted on April (n = 29985) and October (n = 159).
**Figure S2.** Breakfast frequency and the cumulative probabilities of the incidence of weight gain ≥ 10% (a, b) and BMI ≥ 25 kg/$m^2$ (c, d) in male (a, c) and female (b, d) students.
**Figure S3.** Lunch frequency and the cumulative probabilities of the incidence of weight gain ≥ 10% (a, b) and BMI ≥ 25 kg/$m^2$ (c, d) in male (a, c) and female (b, d) students.

**Table S1.** Baseline characteristics, observational period, and outcome incidences of 17,573 male students stratified by breakfast frequency.

| **Breakfast frequency** | **Eating every day** | **Skipping occasionally** | **Skipping ≥ often** | **P** |
|---|---|---|---|---|
| Number | 14,233 | 2275 | 1065 | |
| Baseline characteristics at admission | | | | |
| Age,18 years, n (%) | 9438 (66.3) | 1326 (58.3) | 578 (54.3) | < 0.001 |
| 19 | 4413 (31.0) | 820 (36.0) | 392 (36.8) | |
| 20 | 263 (1.8) | 82 (3.6) | 42 (3.9) | |
| 21–60 | 119 (0.8) | 47 (2.1) | 53 (5.0) | |
| Height, cm | 171.4 ± 5.7 | 171.0 ± 5.7 | 171.0 ± 5.5 | 0.763 |
| Body weight, kg | 63.6 ± 9.4 | 62.8 ± 9.3 | 62.1 ± 9.2 | < 0.001 |
| BMI, kg/m$^2$ | 21.6 ± 2.9 | 21.5 ± 2.9 | 21.2 ± 2.9 | < 0.001 |
| < 25.0 kg/m$^2$, n (%) | 12,657 (88.9) | 2033 (89.4) | 966 (90.7) | 0.181 |
| ≥ 25.0 | 1576 (11.1) | 242 (10.6) | 99 (9.3) | |
| Smokers, n (%) | 45 (0.3) | 35 (1.5) | 17 (1.6) | < 0.001 |
| Drinkers, n (%) | 1156 (8.1) | 317 (13.9) | 185 (17.4) | < 0.001 |
| Sleep duration, < 5 hours, n (%) | 424 (3.0) | 71 (3.1) | 52 (4.9) | < 0.001 |
| 5-6 | 4558 (32.0) | 714 (31.4) | 332 (31.2) | |
| 6-7 | 6951 (48.8) | 1071 (47.1) | 451 (42.3) | |
| 7-8 | 2019 (14.2) | 327 (14.4) | 175 (16.4) | |
| ≥ 8 | 281 (2.0) | 92 (4.0) | 55 (5.2) | |
| Lunch, Eating every day, n (%) | 13,639 (95.8) | 1956 (86.0) | 853 (80.1) | < 0.001 |
| Skipping ≥ occasionally | 594 (4.2) | 319 (14.0) | 212 (19.9) | |
| Dinner, Eating every day, n (%) | 14,115 (99.2) | 2170 (95.4) | 1022 (96.0) | < 0.001 |
| Skipping ≥ occasionally | 118 (0.8) | 105 (4.6) | 43 (4.0) | |
| Dinner time, before 7 PM, n (%) | 2708 (19.0) | 387 (17.0) | 160 (15.0) | < 0.001 |
| 7–9 PM | 9310 (65.4) | 1487 (65.4) | 656 (61.6) | |
| 9–11 PM | 2093 (14.7) | 371 (16.3) | 217 (20.4) | |
| after 11 PM | 122 (0.9) | 30 (1.3) | 32 (3.0) | |
| Observational period and outcome incidences | | | | |
| Observational period (years) | 3.0 ± 0.9 | 3.0 ± 0.9 | 2.9 ± 1.0 | 0.020 |
| Weight gain ≥ 10%, n (%) | 1512 (10.6) | 242 (10.6) | 142 (13.3) | 0.022 |
| IR per 1000 PY (95% CI) | 35.5 (33.7 37.3) | 35.8 (31.6, 40.6) | 45.6 (38.7, 53.8) | |
| BMI ≥ 25 kg/m$^2$, n (%) | 534 (4.2) | 97 (4.8) | 40 (4.1) | 0.508 |
| IR per 1000 PY (95% CI) | 13.8 (12.7, 15.1) | 15.9 (13.0, 19.3) | 13.9 (10.2, 19.0) | |

Mean ± standard deviation; BMI, body mass index; CI, confidence interval, IR, incidence rate; PY, person-year.

**Table S2.** Baseline characteristics, observational period, and incidence of outcomes of 17,573 male students stratified by lunch frequency.

| **Lunch frequency** | **Eating every day** | **Skipping ≥ occasionally** | **P** |
|---|---|---|---|
| Number | 16,448 | 1125 | |
| Baseline characteristics at admission | | | |
| Age,18 years, n (%) | 10,772 (65.5) | 570 (50.7) | < 0.001 |
| 19 | 5171 (31.4) | 454 (40.4) | |
| 20 | 325 (2.0) | 62 (5.5) | |
| 21–60 | 180 (1.1) | 39 (3.5) | |
| Height, cm | 171.3 ± 5.7 | 171.0 ± 5.7 | 0.763 |
| Body weight, kg | 63.4 ± 9.4 | 63.7 ± 10.0 | 0.324 |
| BMI, kg/m$^2$ | 21.6 ± 2.9 | 21.7 ± 3.1 | 0.078 |
| < 25.0 kg/m$^2$, n (%) | 14,682 (89.3) | 974 (86.6) | 0.005 |
| ≥ 25.0 | 1766 (10.7) | 151 (13.4) | |
| Smokers, n (%) | 70 (0.4) | 27 (2.4) | < 0.001 |
| Drinkers, n (%) | 1463 (8.9) | 195 (17.3) | < 0.001 |
| Sleep duration, < 5 hours, n (%) | 484 (2.9) | 63 (5.6) | < 0.001 |
| 5-6 | 5207 (31.7) | 397 (35.3) | |
| 6-7 | 8010 (48.7) | 463 (41.2) | |
| 7-8 | 2369 (14.4) | 152 (13.5) | |
| ≥ 8 | 378 (2.3) | 50 (4.4) | |
| Breakfast, Eating every day, n (%) | 13,639 (82.9) | 594 (52.8) | < 0.001 |
| Skipping occasionally | 1956 (11.9) | 319 (28.4) | |
| Skipping ≥ often | 853 (5.2) | 212 (18.8) | |
| Dinner, Eating every day, n (%) | 16,327 (99.3) | 980 (87.1) | < 0.001 |
| Skipping ≥ occasionally | 121 (0.7) | 145 (12.9) | |
| Dinner time, before 7 PM, n (%) | 3078 (18.7) | 177 (15.7) | <0.001 |
| 7–9 PM | 10,747 (65.3) | 706 (62.8) | |
| 9–11 PM | 2466 (15.0) | 215 (19.1) | |
| after 11 PM | 157 (1.0) | 27 (2.4) | |
| Observational period and outcome incidences | | | |
| Observational period (years) | 3.0 ± 0.9 | 3.0 ± 0.9 | 0.904 |
| Weight gain ≥ 10%, n (%) | 1760 (10.7) | 136 (12.1) | 0.146 |
| IR per 1000 PY (95% CI) | 35.8 (34.2, 37.5) | 40.5 (34.2, 47.9) | |
| BMI ≥ 25 kg/m$^2$, n (%) | 621 (4.2) | 50 (5.1) | 0.177 |
| IR per 1000 PY (95% CI) | 13.9 (12.9, 15.0) | 17.0 (12.9, 22.5) | |

Mean ± standard deviation; BMI, body mass index; CI, confidence interval, IR, incidence rate; PY, person-year.

**Table S3.** Baseline characteristics, observational period, and outcome incidences of 8860 female students stratified by breakfast frequency.

| **Breakfast frequency** | **Eating every day** | **Skipping occasionally** | **Skipping ≥ often** | **P** |
|---|---|---|---|---|
| Number | 7793 | 782 | 285 | |
| Baseline characteristics at admission | | | | |
| Age,18 years, n (%) | 5832 (74.8) | 536 (68.5) | 169 (59.3) | < 0.001 |
| 19 | 1784 (22.9) | 192 (24.6) | 76 (26.7) | |
| 20 | 111 (1.4) | 28 (3.6) | 21 (7.4) | |
| 21–60 | 66 (0.8) | 26 (3.3) | 19 (6.7) | |
| Height, cm | 158.4 ± 5.2 | 158.2 ± 5.1 | 158.7 ± 5.6 | 0.250 |
| Body weight, kg | 51.6 ± 6.8 | 51.0 ± 6.9 | 51.5 ± 7.0 | 0.088 |
| BMI, $kg/m^2$ | 20.5 ± 2.4 | 20.4 ± 2.5 | 20.4 ± 2.4 | 0.137 |
| < 25.0 $kg/m^2$, n (%) | 7450 (95.6) | 740 (94.6) | 275 (96.5) | 0.334 |
| ≥ 25.0 | 343 (4.4) | 42 (5.4) | 10 (3.5) | |
| Smokers, n (%) | 7 (0.1) | 4 (0.5) | 2 (0.7) | 0.001 |
| Drinkers, n (%) | 309 (4.0) | 67 (8.6) | 42 (14.7) | < 0.001 |
| Sleep duration, < 5 hours, n (%) | 212 (2.7) | 35 (4.5) | 17 (6.0) | < 0.001 |
| 5-6 | 2790 (35.8) | 276 (35.3) | 93 (32.6) | |
| 6-7 | 3753 (48.2) | 341 (43.6) | 115 (40.4) | |
| 7-8 | 951 (12.2) | 112 (14.3) | 46 (16.1) | |
| ≥ 8 | 87 (1.1) | 18 (2.3) | 14 (4.9) | |
| Lunch, Eating every day, n (%) | 7546 (96.8) | 679 (86.8) | 226 (79.3) | < 0.001 |
| Skipping ≥ occasionally | 247 (3.2) | 103 (13.2) | 59 (20.7) | |
| Dinner, Eating every day, n (%) | 7538 (96.7) | 707 (90.4) | 257 (90.2) | < 0.001 |
| Skipping ≥ occasionally | 255 (3.3) | 75 (9.6) | 28 (9.8) | |
| Dinner time, before 7 PM, n (%) | 2100 (26.9) | 137 (17.5) | 51 (17.9) | < 0.001 |
| 7–9 PM | 4917 (63.1) | 519 (66.4) | 175 (61.4) | |
| 9–11 PM | 746 (9.6) | 121 (15.5) | 53 (18.6) | |
| after 11 PM | 30 (0.4) | 5 (0.6) | 6 (2.1) | |
| Observational period and outcome incidences | | | | |
| Observational period (years) | 3.0 ± 1.0 | 2.9 ± 1.0 | 2.9 ± 1.1 | 0.064 |
| Weight gain ≥ 10%, n (%) | 1338 (17.2) | 138 (17.6) | 42 (14.7) | 0.519 |
| IR per 1000 PY (95% CI) | 58.1 (55.0, 61.3) | 61.4 (51.9, 72.5) | 50.4 (37.3, 68.3) | |
| BMI ≥ 25 $kg/m^2$, n (%) | 236 (3.2) | 17 (2.3) | 13 (4.7) | 0.134 |
| IR per 1000 PY (95% CI) | 10.0 (8.8, 11.4) | 7.5 (4.7, 12.1) | 15.3 (8.9, 26.4) | |

Mean ± standard deviation; BMI, body mass index; CI, confidence interval, IR, incidence rate; PY, person-year.

**Table S4.** Baseline characteristics, observational period, and outcome incidences of 8860 female students stratified by lunch frequency.

| **Lunch frequency** | **Eating every day** | **Skipping ≥ occasionally** | **P** |
|---|---|---|---|
| Number | 8451 | 409 | |
| Baseline characteristics at admission | | | |
| Age,18 years, n (%) | 6283 (74.3) | 254 (62.1) | < 0.001 |
| 19 | 1933 (22.9) | 119 (29.1) | |
| 20 | 140 (1.7) | 20 (4.9) | |
| 21-60 | 95 (1.1) | 16 (3.9) | |
| Height, cm | 158.4 ± 5.2 | 158.2 ± 5.6 | 0.250 |
| Body weight, kg | 51.5 ± 6.8 | 51.7 ± 6.9 | 0.483 |
| BMI, kg/m$^2$ | 20.5 ± 2.4 | 20.7 ± 2.4 | 0.221 |
| < 25.0 kg/m$^2$, n (%) | 8078 (95.6) | 387 (94.6) | 0.356 |
| ≥ 25.0 | 373 (4.4) | 22 (5.4) | |
| Smokers, n (%) | 11 (0.1) | 2 (0.5) | 0.064 |
| Drinkers, n (%) | 370 (4.4) | 48 (11.7) | < 0.001 |
| Sleep duration, < 5 hours, n (%) | 241 (2.9) | 23 (5.6) | < 0.001 |
| 5-6 | 3011 (35.6) | 148 (36.2) | |
| 6-7 | 4045 (47.9) | 164 (40.1) | |
| 7-8 | 1045 (12.4) | 64 (15.6) | |
| ≥ 8 | 109 (1.3) | 10 (2.4) | |
| Breakfast, Eating every day, n (%) | 7546 (89.3) | 247 (60.4) | < 0.001 |
| Skipping occasionally | 679 (8.0) | 103 (25.2) | |
| Skipping ≥ often | 226 (2.7) | 59 (14.4) | |
| Dinner, Eating every day, n (%) | 8178 (96.8) | 324 (79.2) | < 0.001 |
| Skipping ≥ occasionally | 273 (3.2) | 85 (20.8) | |
| Dinner time, before 7 PM, n (%) | 2204 (26.1) | 84 (20.5) | 0.006 |
| 7–9 PM | 5347 (63.3) | 264 (64.5) | |
| 9–11 PM | 863 (10.2) | 57 (13.9) | |
| after 11 PM | 37 (0.4) | 4 (1.0) | |
| Observational period and outcome incidences | | | |
| Observational period (years) | 2.9 ± 1.0 | 2.9 ± 1.0 | 0.412 |
| Weight gain ≥ 10%, n (%) | 1452 (17.2) | 66 (16.1) | 0.583 |
| IR per 1000 PY (95% CI) | 58.2 (55.3, 61.3) | 55.5 (43.6, 70.6) | |
| BMI ≥ 25 kg/m$^2$, n (%) | 250 (3.1) | 16 (4.1) | 0.253 |
| IR per 1000 PY (95% CI) | 9.8 (8.7, 11.1) | 13.6 (8.4, 22.3) | |

Mean ± standard deviation; BMI, body mass index; CI, confidence interval, IR, incidence rate; PY, person-year.

**Table S5.** Baseline characteristics of 212 male students with baseline dinner frequency of skipping ≥ occasionally who underwent an annual health checkup 1 year after admission.

| **Dinner frequency 1 year after admission** | **Eating every day** | **Skipping ≥ occasionally** | **P** |
|---|---|---|---|
| Number | 164 | 48 | |
| Baseline characteristics at admission | | | |
| Age, 18 years, n (%) | 64 (39.0) | 23 (47.9) | 0.330 |
| 19 | 79 (48.2) | 19 (39.6) | |
| 20 | 14 (8.5) | 2 (4.2) | |
| 21-60 | 7 (4.3) | 4 (8.3) | |
| Height, cm | 171.0 ± 5.6 | 170.7 ± 6.1 | 0.770 |
| Body weight, kg | 66.2 ± 10.8 | 72.1 ± 18.8 | 0.007 |
| BMI, kg/m$^2$ | 22.6 ± 3.5 | 24.6 ± 5.8 | 0.004 |
| < 25.0 kg/m$^2$, n (%) | 133 (81.1) | 31 (64.6) | 0.016 |
| ≥ 25.0 | 31 (18.9) | 17 (35.4) | |
| Smokers, n (%) | 3 (1.8) | 4 (8.3) | 0.027 |
| Drinkers, n (%) | 41 (25.0) | 6 (12.5) | 0.067 |
| Sleep duration, < 5 hours, n (%) | 10 (6.1) | 5 (10.4) | 0.624 |
| 5-6 | 64 (39.0) | 20 (41.7) | |
| 6-7 | 65 (39.6) | 14 (29.2) | |
| 7-8 | 21 (12.8) | 7 (14.6) | |
| ≥ 8 | 4 (2.4) | 2 (4.2) | |
| Breakfast, Eating every day, n (%) | 74 (45.1) | 23 (47.9) | 0.174 |
| Skipping occasionally | 58 (35.4) | 21 (43.8) | |
| Skipping ≥ often | 32 (19.5) | 4 (8.3) | |
| Lunch, Eating every day, n (%) | 78 (47.6) | 24 (50.0) | 0.766 |
| Skipping ≥ occasionally | 86 (52.4) | 24 (50.0) | |
| Dinner time, before 7 PM, n (%) | 32 (19.5) | 12 (25.0) | 0.594 |
| 7–9 PM | 88 (53.7) | 27 (56.3) | |
| 9–11 PM | 35 (21.3) | 8 (16.7) | |
| after 11 PM | 9 (5.5) | 1 (2.1) | |

Mean ± standard deviation; BMI, body mass index

**Table S6.** Baseline characteristics of 287 female students with baseline dinner frequency of skipping ≥ occasionally who underwent an annual health checkup 1 year after admission.

| **Dinner frequency 1 year after admission** | **Eating every day** | **Skipping ≥ occasionally** | **P** |
|---|---|---|---|
| Number | 171 | 116 | |
| Baseline characteristics at admission | | | |
| Age,18 years, n (%) | 100 (58.5) | 65 (56.0) | 0.971 |
| 19 | 56 (32.7) | 41 (35.3) | |
| 20 | 8 (4.7) | 5 (4.3) | |
| 21-60 | 7 (4.1) | 5 (4.3) | |
| Height, cm | 158.6 ± 5.4 | 159.0 ± 4.9 | 0.446 |
| Body weight, kg | 53.1 ± 7.3 | 53.2 ± 7.0 | 0.887 |
| BMI, kg/m$^2$ | 21.1 ± 2.6 | 21.0 ± 2.3 | 0.763 |
| < 25.0 kg/m$^2$, n (%) | 158 (92.4) | 107 (92.2) | 0.961 |
| ≥ 25.0 | 13 (7.6) | 9 (7.8) | |
| Smokers, n (%) | 0 (0.0) | 2 (1.7) | 0.085 |
| Drinkers, n (%) | 19 (11.1) | 21 (18.1) | 0.093 |
| Sleep duration, < 5 hours, n (%) | 9 (5.3) | 7 (6.0) | 0.494 |
| 5-6 | 63 (36.8) | 42 (36.2) | |
| 6-7 | 66 (38.6) | 45 (38.8) | |
| 7-8 | 31 (18.1) | 17 (14.7) | |
| ≥ 8 | 2 (1.2) | 5 (4.3) | |
| Breakfast, Eating every day, n (%) | 117 (68.4) | 86 (74.1) | 0.140 |
| Skipping occasionally | 42 (24.6) | 18 (15.5) | |
| Skipping ≥ often | 12 (7.0) | 12 (10.3) | |
| Lunch, Eating every day, n (%) | 130 (76.0) | 92 (79.3) | 0.514 |
| Skipping ≥ occasionally | 41 (24.0) | 24 (20.7) | |
| Dinner time, before 7 PM, n (%) | 45 (26.3) | 45 (38.8) | 0.074 |
| 7–9 PM | 101 (59.1) | 54 (46.6) | |
| 9–11 PM | 25 (14.6) | 16 (13.8) | |
| after 11 PM | 0 (0.0) | 1 (0.9) | |

Mean ± standard deviation; BMI, body mass index

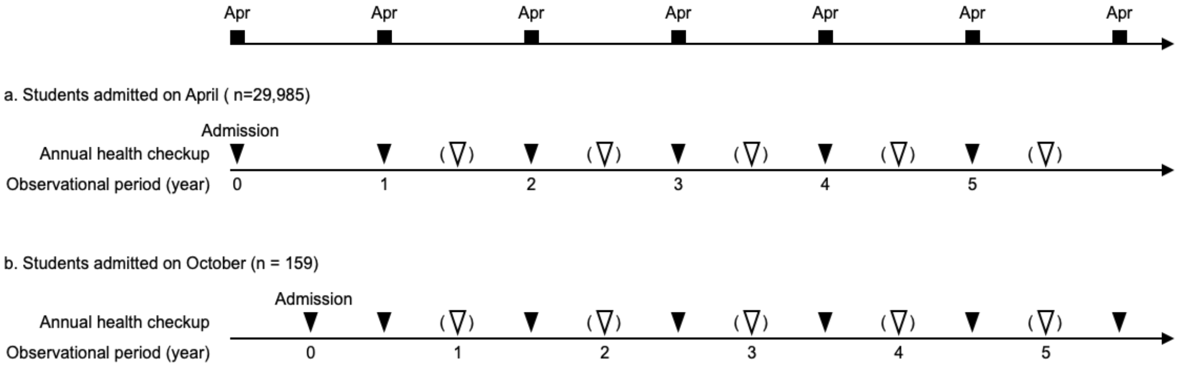


**Figure S1**. Annual health checkups during the observational period in students admitted on April (n = 29,985) and October (n = 159). The vast majority of students underwent their annual health checkups every April after admission (black triangle). The university provided alternative health checkups on Octobers (white triangle) for students who could not underwent their health checkups on April.

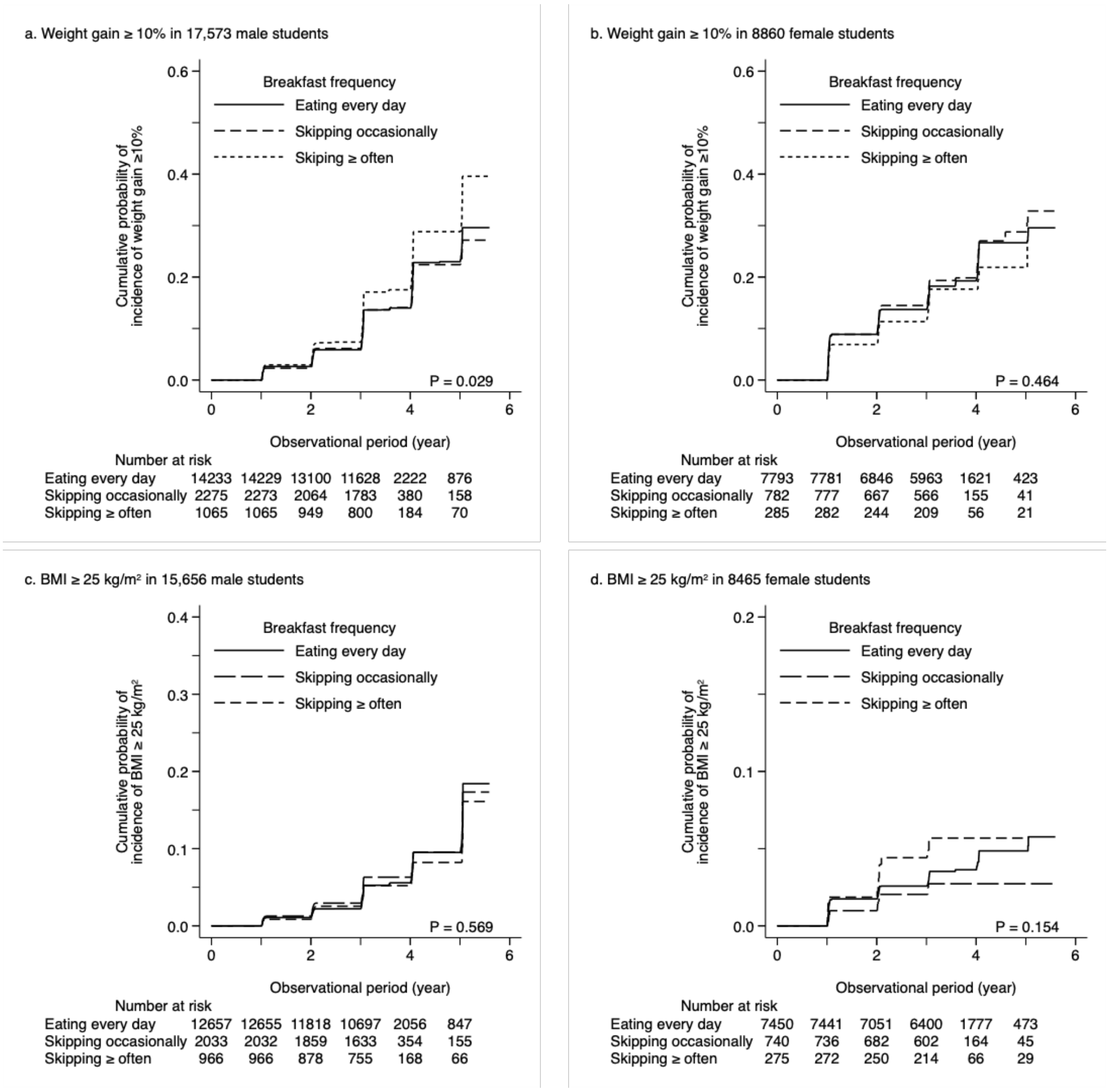


**Figure S2.** Breakfast frequency and the cumulative probabilities of the incidence of weight gain ≥ 10% (a, b) and BMI ≥ 25 kg/m$^2$ (c, d) in male (a, c) and female (b, d) students. BMI, body mass index.

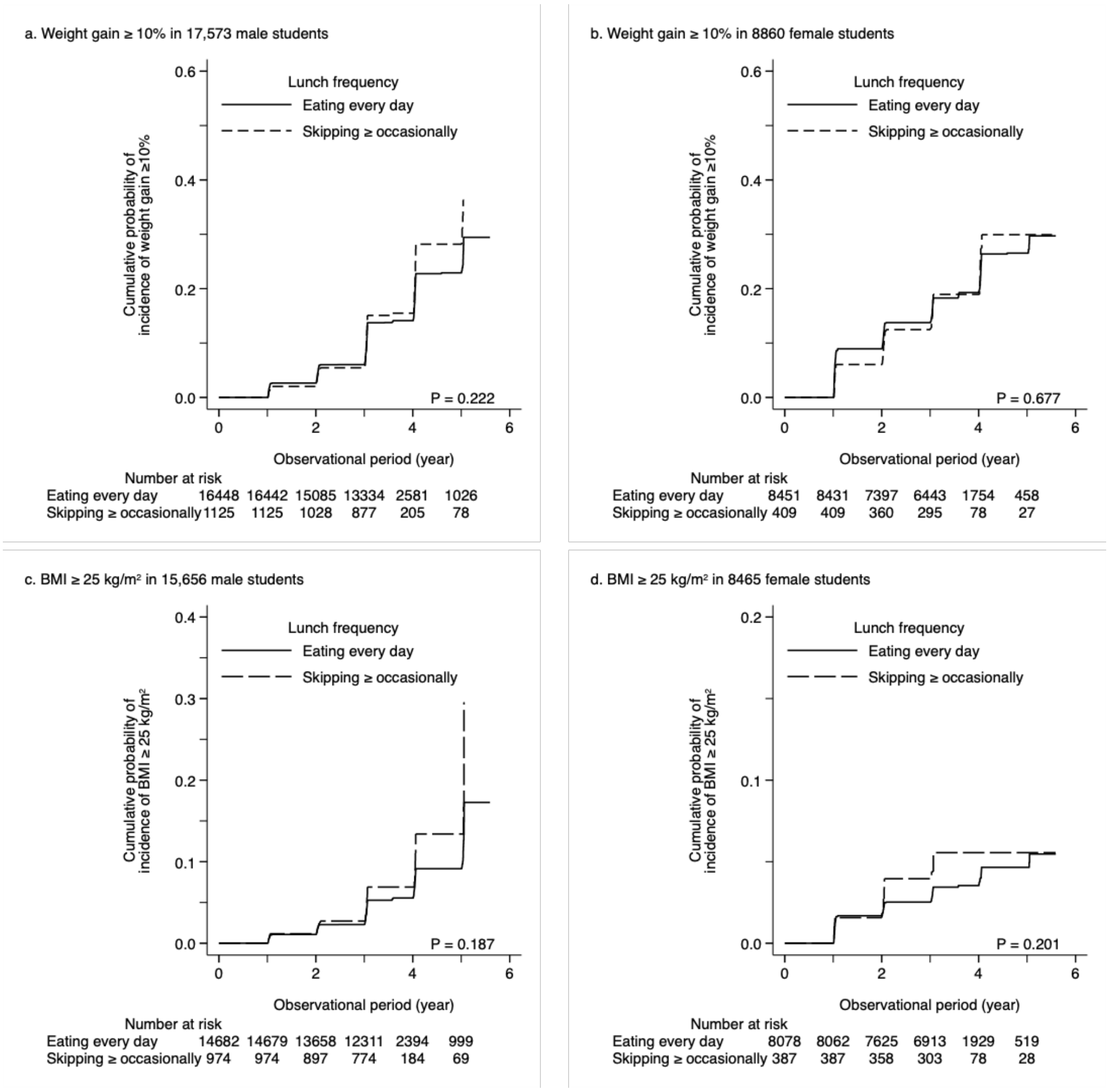


**Figure S3**. Lunch frequency and the cumulative probabilities of the incidence of weight gain ≥ 10% (a, b) and BMI ≥ 25 kg/m$^2$ (c, d) in male (a, c) and female (b, d) students. BMI, body mass index.
